# Reintegration of Ebola Survivors into Their Communities — Firestone District, Liberia, 2014

**Published:** 2014-12-19

**Authors:** M. Allison Arwady, Edmundo L. Garcia, Benedict Wollor, Lyndon G. Mabande, Erik J. Reaves, Joel M. Montgomery

**Affiliations:** 1 Epidemic Intelligence Service, CDC; 2 Firestone Liberia, Inc.; 3 Division of Global Health Protection-Kenya, CDC

The current Ebola virus disease (Ebola) epidemic in West Africa is unprecedented in size and duration (1). Since the outbreak was recognized in March 2014, the World Health Organization (WHO) has reported 17,145 cases with 6,070 deaths, primarily in Guinea, Liberia, and Sierra Leone (2). Combined data show a case-fatality rate of approximately 70% in patients with a recorded outcome (3); a 30% survival rate means that thousands of patients have survived Ebola. An important component of a comprehensive Ebola response is the reintegration of Ebola survivors into their communities.

Firestone Liberia, Inc. (Firestone) is a large rubber plantation operator in the Firestone District of central Liberia that serves the health needs of approximately 80,000 persons, including employees, their dependents, and retirees, as well as other persons in the district. Firestone District is made up of many small communities, some associated with specified work areas. In April 2014, following the first Ebola case diagnosed in the district, the company established a comprehensive Ebola response, including the management of patients in a dedicated Ebola treatment unit (ETU) (4). Contacts who had a high-risk exposure, such as sharing a household with a confirmed Ebola patient, were encouraged to enter voluntary quarantine in dedicated facilities (converted schools) for 21 days. Firestone offered this voluntary quarantine to nonemployees as well as employees. Persons in voluntary quarantine were closely monitored and at the first sign of symptoms were transferred to the ETU for testing and care. To prepare communities for the return of Ebola survivors and minimize potential stigmatization, Firestone established a survivor reintegration program.

Survivors of past Ebola epidemics have reported substantial negative psychosocial impacts. In one study, 35% of survivors reported feeling rejected by society, including by family, friends, and neighbors (5). Survivors often face stigma, income loss, and grief, particularly if friends and family members have died; in addition, many of their possessions have been destroyed to prevent disease transmission (6). Some family members concerned about infection have been reluctant to accept orphaned children (7). However, survivors also have long-lasting antibodies to the circulating Ebola virus strain that could confer immunity to subsequent infection with the same strain (8). Survivors might be able to provide care to infected persons, although they should follow infection control protocols, including use of appropriate personal protective equipment that is recommended for all persons providing care to Ebola patients. Some survivors have donated plasma to other Ebola patients, although the benefit of passive immunotherapy is, as yet, unproven (9). Survivors also can play important roles in educating communities about Ebola, particularly in areas with high infection rates, where fear might prevent ill persons from seeking medical care. They can offer hope that survival is possible if medical care is obtained during the early stages of infection (10).

## Epidemiologic Characteristics of Survivors

During August 1–November 1, 2014, 33 Ebola patients (30 laboratory-confirmed using a real-time reverse transcription–polymerase chain reaction assay at the Liberian Institute of Biomedical Research) died in the Firestone ETU. But during the same period, 22 survivors who had laboratory-confirmed Ebola were discharged from the ETU after symptom resolution and negative follow-up Ebola testing, yielding a survival rate of 42%. In the ETU, 5 days after all of a laboratory-confirmed Ebola patient’s symptoms had resolved, blood was retested, using the same procedure as before. If the repeat sample was negative, the survivor was transferred to a recovery room in the ETU and remained there for 3 more days before ETU discharge. This period was used to educate and counsel the survivor and to make preparations with the survivor’s community for a return home.

Thirteen (60%) of the 22 Ebola survivors were Firestone employees or dependents, six were retiree dependents, and three had no connection to Firestone. The mean age of survivors was 23 years (range = 8 months–54 years), and they were significantly younger (p = 0.003, by Student’s t-test) than nonsurvivors, whose mean age was 38 years (range = 4 years–81 years). Six (27%) survivors were children aged <13 years, six (27%) were teens aged 13–17 years, and the remaining 10 were adults aged ≥21 years. Twelve (55%) were female. Ten survivors had at least one other family member who also was a survivor (two families had three survivors; two had two survivors). Before reintegration, the 22 survivors had been in isolation and treatment at the Firestone ETU for a mean of 16 days (range = 9–23 days).

Fourteen (64%) of the 22 survivors were being followed as contacts of known patients with Ebola when they became ill, and 12 (86%) of these had been in voluntary quarantine for a mean of 7 days (range = 1–13 days) before symptom onset. As of November 1, a total of 250 contacts from 63 families had entered voluntary quarantine, 167 (67%) of whom were Firestone employees or dependents.

## Survivor Reintegration Process

Plans for reintegration into the community begin before the survivor leaves the ETU, with a goal of helping the family and home community accept the survivor’s return. For 1 or 2 days before a survivor is released from the ETU, Firestone’s reintegration team travels to the survivor’s home and meets with neighbors and community leaders to discuss the plan to bring the survivor home. At this meeting, the team educates the community about Ebola transmission, emphasizing that survivors are no longer ill and have been declared free from Ebola. The team encourages and answers questions and addresses community concerns to help ensure that survivors are welcomed and not stigmatized. If the survivor is a child, the team also ensures that appropriate guardians have been identified and that the child will be able to continue attending school. The team and community then plan a program to receive the survivor back into the community.

**FIGURE 1:**
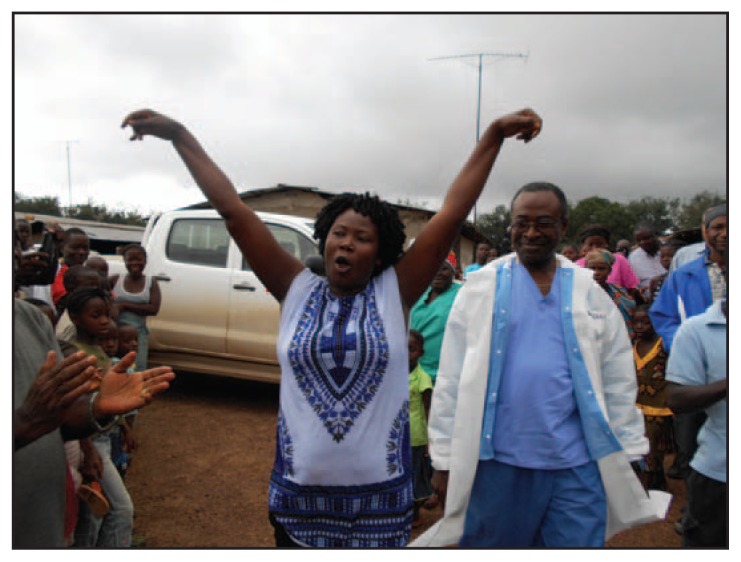
Ebola survivor, accompanied by medical director, being welcomed by her community — Firestone District, Liberia, 2014

On the day of reintegration, Firestone’s medical director, the ETU coordinator, and other medical staff members bring the survivor to the community, accompanied by the reintegration team, radio station personnel, and clergy (Figure 1). If the survivor is a Firestone employee or an employee’s dependent, work supervisors and teammates also attend. Representatives from the Ministry of Health and Social Welfare are also invited, and community members decorate the survivor’s home with traditional palm leaves to signify the festive occasion.

The formal program begins with prayers and a praise and worship session, led by the community and clergy members. A local community leader makes opening remarks and officially welcomes the survivor home. The Firestone Health Services medical director speaks about the survivor’s recovery and about the importance of seeking immediate medical attention when one gets sick. Representatives from the county health team and the Ministry of Health and Social Welfare emphasize ongoing education and response efforts. The tone throughout the event is celebratory; holding a separate pre-integration meeting in the preceding days ensures that community concerns and questions have been addressed before the survivor’s arrival.

The survivor is given an opportunity to speak, and many adults choose to describe their recent care in the ETU. These first-hand survivor accounts have been powerful tools to help dispel misconceptions and fears about what happens in an ETU. The program is broadcast live on the radio and replayed several times after the occasion. In some cases, messages from survivors have been reused in radio programs devoted to Ebola education and awareness.

**FIGURE 2:**
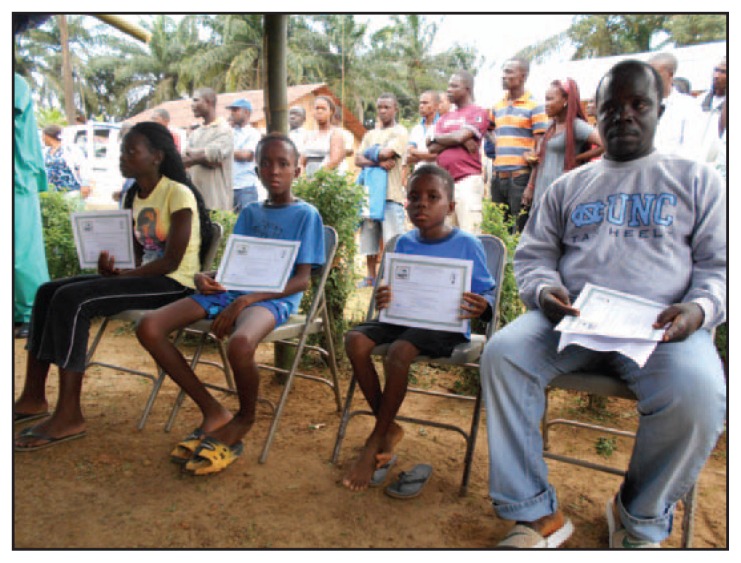
Ebola survivors, three orphans and their uncle, receiving Certificate of Medical Clearance as part of the Firestone Ebola Survivor Reintegration Program — Firestone District, Liberia, 2014

**FIGURE 3:**
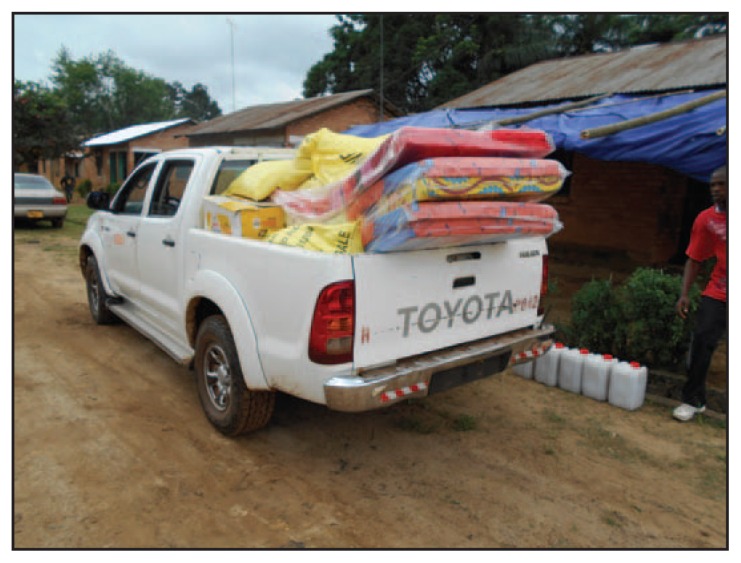
Truck transporting solidarity kits containing essential supplies for Ebola survivors returning home — Firestone District, Liberia, 2014

The medical director presents the survivor with a laminated Certificate of Medical Clearance, declaring that the individual is free from Ebola (Figure 2). The back of the certificate includes reminders for survivors, advising temporary abstinence from sex and covering ways the survivor might use recovery to benefit others (e.g., “do not donate blood until you feel strong and are advised by your doctor,” and “help educate others about Ebola and share your experiences freely”). Each survivor also receives a solidarity kit, which includes a new mattress, bedding, towels, an insecticide-treated mosquito net, soaps and toiletries, a 50-kg bag of rice, 3 gallons (11 liters) of cooking oil, toys for children, clothing, and cash for food and personal necessities (Figure 3).

After the reintegration ceremony, the physician-led Firestone medical team visits all survivors at home every week for 3 months, both for a clinical checkup and to provide social and psychological support. One month after ETU discharge, blood is drawn for a follow-up blood chemistry analysis.

No major reintegration problems have occurred to date; one survivor (the wife of a Firestone employee) who had not been a full-time resident in Firestone District before her illness reported some initial social exclusion but gained acceptance over time. None of the other survivors, including those who were not Firestone employees or dependents, reported major problems reintegrating into their communities. All who were employed have returned to work, all orphans continue to live with their designated guardians, and arrangements have been made to ensure that all children are able to resume schooling once schools reopen (all schools remain closed by government decree because of the ongoing epidemic). There have been no housing issues, attacks on survivors, or other episodes of community unrest. The reintegration ceremonies continue to be well-attended by dozens of community members, and the two-stage meeting approach by the reintegration team has ensured celebratory rather than confrontational events.

Although official reintegration programs do not allow for anonymity and can raise questions of survivor privacy, in the small communities in the Firestone District a person’s status as an Ebola patient is already widely known. Formal reintegration programs legitimize family and community member concerns regarding Ebola transmission risks, offer opportunities for continued education, and provide an important first step in the necessary psychosocial support for survivors. When these programs are made public, they can help dispel rumors, provide hope, and encourage community members to report suspected Ebola cases or seek care early, which can, in turn, decrease transmission and increase survival among those with infection.
